# Handedness throughout the lifespan: cross-sectional view on sex differences as asymmetries change

**DOI:** 10.3389/fpsyg.2014.01556

**Published:** 2015-01-14

**Authors:** Mukundhan Sivagnanasunderam, Dave A. Gonzalez, Pamela J. Bryden, Gordon Young, Amanda Forsyth, Eric A. Roy

**Affiliations:** ^1^Department of Kinesiology, University of WaterlooWaterloo, ON, Canada; ^2^Department of Kinesiology, Wilfrid Laurier UniversityWaterloo, ON, Canada

**Keywords:** lifespan, handedness, manual asymmetries, pegboard, sex differences

## Abstract

Manual asymmetries has been studied by many researchers, however contradictory findings still exist as to whether preferred manual asymmetries increases with age or do we become more ambidextrous. Recently it was shown that perhaps there is a third option, that there is no increase or decrease in laterality but rather preferred manual asymmetries remains consistent throughout adulthood. Another related finding is that females appear to have an advantage in some handedness tasks, such as the Grooved Pegboard. When a larger pegboard is used, sex differences may reverse as males may perform better when larger pegs and a larger trajectory are required. However, it is not fully understood if these sex differences arise from an early age and continue throughout life. Therefore, we sought to explore sex differences in preferred hand dominance throughout the lifespan. In order to explore preferred hand dominance during the lifespan we examined 76 children (19.4–5 year olds, 12 female, *M*_age_ = 4.73; 34.6–8 year olds, 12 female, *M*_age_ = 6.97; 23.9–12 year olds, 14 female, *M*_age_ = 10.83) in Experiment 1 and 35 healthy young right-handed adults (15 female, *M*_age_ = 20.91) and 37 healthy older right-handed adults (20 female, *M*_age_ = 72.3) in Experiment 2. Individuals were tested using a standard size (small) and modified Grooved Pegboard (larger pegboard). Our study demonstrates that hand asymmetries are present early in life (children 4–5 years old) at that these differences attenuate as a function of age until adulthood (Experiment 1). Furthermore, our results demonstrate that as we age (Experiment 2), asymmetries may increase (small and large pegboards), decrease (Annett), or stay the same (finger tapping). As well we demonstrated that the sex differences could not be entirely accounted for by hand size. Therefore, asymmetries as regard to the aging process, seems to be task specific which may account for the conflicting findings in research.

## Introduction

Manual asymmetries are the differences in performance abilities between the preferred and non-preferred hand (Corey et al., 2001). One of the leading hypotheses is that manual asymmetries exist because of an individual's continued reliance on their preferred hand throughout their lifespan (Peters, 1976; Provins, 1997). For example, approximately 90% of the adult population prefers to use their preferred hand for a myriad of everyday tasks, such as writing, holding a cup, brushing their teeth, and other one-handed manual tasks (Brown et al., 2006). However, the progression and direction of hand preference varies as a person ages: children aged 3 and under are considered “mixed-handed”; adolescent (10–12 years of age) individuals seemingly preferring to exclusively use their dominant hand for various tasks (Gesell and Ames, 1947; Ittyerah, 2013; Gooderham and Bryden, 2014); while adults appear to rely less on the preferred hand (Gooderham and Bryden, 2014). Furthermore, there have been conflicting findings as to whether asymmetries increase (e.g., Weller and Latimer-Sayer, 1985), decline (e.g., Kalisch et al., 2006), or remain constant (e.g., Chua et al., 1995; Francis and Spirduso, 2000; Cabeza, 2002; Hausmann et al., 2003; Przybyla and Sainburg, 2010) throughout the rest of our lives. Therefore, this study endeavors to provide an examination of the changes in the strength of manual asymmetries throughout the lifespan by comparing children, adults, and older adults. Secondly this study investigates how sex differences may impact manual asymmetries.

One method of determining laterality throughout the lifespan is to compare hand preference and performance of children, adults, and older adults. A study conducted by Carlier et al. (2006) on children aged 3–10 clearly demonstrates an increased reliance on the preferred hand as a function of age. In this study cards were placed in a semi-circle in front of the participant; with three cards being placed on the right side and three on the left. The authors noticed that younger children aged 3–4 reached across their body/midline to grasp the cards less often than children aged 7 and above. Reaching across the midline is considered a less efficient biomechanical movement as the hand has to travel further compared to using the hand on the same side, which also demonstrates an individual's reliance on their preferred hand to accomplish various tasks. Therefore, the number of children reaching with their preferred hand (right hand) to grasp cards on their left side increased with age, suggesting that younger children (aged 3–4) may produce more biomechanically efficient movements or are less dependent on their preferred hand (Carlier et al., 2006). At an older age biomechanical efficiency is seemingly replaced with hand preference, as children aged 8–10 prefer to rely on their preferred hand for reaching (Gesell and Ames, 1947; McManus et al., 1988; Carlier et al., 2006; Ittyerah, 2013).

Therefore, hand preference seems to increase and perhaps peak around 8–10 years old (Carlier et al., 2006), with preferential hand reaching across the midline decreasing in adulthood (e.g., Bryden and Roy, 2006). However, research regarding how we progressively age after adulthood demonstrates conflicting findings regarding changes in manual asymmetries. There exist at least two partially conflicting models that endeavor to explain the direction and degree of manual asymmetries with respect to aging (Weller and Latimer-Sayer, 1985; Cabeza, 2002; Hausmann et al., 2003) with one hypothesis based on the hemi-aging model. The hemi-aging model states that the advantage seen for the preferred hand's performance would become more pronounced, with a person essentially reverting back to their performance during adolescent years (Brown and Jaffe, 1975; Weller and Latimer-Sayer, 1985; Albert, 1988). Evidence for the hemi-aging model is based on the decline in performance IQ (Wechsler Adult Intelligence Scale) of older adults (Goldstien and Shelley, 1981), which incorporates subtests that have a speed component (unlike the verbal IQ). These findings formed the crux of hemi-aging model and indicated that the right hemisphere aged more rapidly than the left and also suggested a more rapid decline in left hand motor performance (Meudell and Greenhalgh, 1987). To test this hypothesis, Weller and Latimer-Sayer (1985) used a standardized Grooved Pegboard (Federal Security Agency, 1949), which is a visuomotor task that measures motor performance of both hands. They found that abilities typically associated with the right hemisphere were affected more by aging and that the motor performance of the left hand, which is controlled by the right hemisphere, declined to a greater degree than the right hand (Weller and Latimer-Sayer, 1985).

Although the hemi-aging model was quite popular, more recently research has produced an alternative view. The second hypothesis is called the hemispheric asymmetry reduction in older adults, or HAROLD Model and is based on the results of functional neuroimaging of patterns of activation during cognitive and motor tasks in younger and older adults (Cabeza, 2001). Previous research utilizing unimanual motor tasks like finger tapping and hand grip task (grasping at percentage of peak grip strength) have revealed a more symmetric hemispheric activation (Mattay et al., 2002; Ward and Frackowiak, 2003; Rowe et al., 2006). A study employing a unimanual reaching task involving older adults (60–80 years old) discovered that there were smaller asymmetries in motor performance between the left and right hands of older adults compared to younger adults (Przybyla et al., 2011). Furthermore, results revealed that older adults using their non-preferred hand generated straighter trajectories (suggests an efficient movement) much like their preferred hand, compared to younger adults who tended to have larger hand path curvatures (suggests less efficient movement) when using their non-preferred hand (Przybyla et al., 2011). Additionally, there were no differences in accuracy between the preferred and non-preferred hand in older adults, but younger adults were more accurate with their preferred hand (Przybyla et al., 2011). A more contemporary hypothesis, similar to the conclusions of the HAROLD model is based on use dependent plasticity and states that as individuals age the performance and ability of the preferred hand will decrease, relative to its non-preferred counterpart simply because of an inactive lifestyle and less usage (Kalisch et al., 2006). The result of a more sedentary lifestyle and underutilization of the preferred hand made the performance differences between the preferred and non-preferred hand less pronounced as individuals aged and resulted in an overall decrease in asymmetries (Kalisch et al., 2006). It should be noted that unlike the HAROLD model where the motor performance of the non-preferred hand improves, this model demonstrates a decline in the performance of the preferred hand as demonstrated using task such as line tracing, aiming, and tapping (Kalisch et al., 2006).

Although aging is an important factor that impacts hand performance, Bornstein (1985) have also demonstrated that females were consistently faster than men on Grooved Pegboard tests, with other researchers replicating these findings (e.g., Ruff and Parker, 1993; Schmidt et al., 2000; Bryden and Roy, 2006). Despite the various studies demonstrating that women perform better than men on the Grooved Pegboard task, there is still a limited understanding as to why this occurs. Researchers have noticed that both men and women with larger fingers had trouble grasping the pegs, which would have negatively affected (i.e., slow down) performance (Peters et al., 1990). Peters et al. (1990) demonstrated that differences in performance between men and women dissipated once finger size was accounted for. To further explore the role of finger size Peters and Campagnaro's (1996) subsequent study had their participants use tweezers to manipulate the pegs and discovered that the performance differences between sexes were negligible. This hypothesis is also supported by the findings of Kilshaw and Annett (1983) as using a larger pegboard revealed no or little differences between the sexes. Therefore, the role of sex differences should be investigated across the lifespan.

Therefore, the purpose of these studies was to examine how the direction and degree or strength of manual asymmetries are affected as a function of age in performance measures (i.e., movement time) and how sex plays a role. We hypothesized that since youngest children would be mixed handed, the performance measures between the hands would not be different. As the age of the participants increased, so would the reliance on the preferred hand and therefore performance with the preferred hand would be better as it is used more. However, the reliance on the preferred hand would decrease once adults were tested (see Gooderham and Bryden, 2014), and performance differences would decrease for older adults, as they would revert to a childhood handedness pattern. Regarding sex, we predict that females will perform better than males, and these differences will dissipate after hand size is accounted.

## Experiment 1

We sought to explore how sex differences may impact asymmetries during the childhood ages by manipulating the size of the pegboards utilized, as well as the corresponding peg size. The specific hypotheses for Experiment 1 were that we would see an interaction between the hand used and age group, and that sex differences would disappear when using the larger pegboard. Specifically, we believed that the youngest children would show smaller differences in performance between preferred and non-preferred hands due to the constant usage of both hands in everyday tasks. Furthermore, children aged 10–12 would demonstrate the largest difference between hand performance, as they tend to rely on their preferred hand (Carlier et al., 2006; Rezaee et al., 2010; Gooderham and Bryden, 2014). We hypothesized that adults would have smaller differences between the hands compared to the children aged 10–12. We also hypothesized that these findings would be seen irrespective of the task used. Thirdly we hypothesized that females would perform better than males (see Peters and Campagnaro's, 1996), but only for the smaller pegboard. Sex differences would disappear when the larger pegboard is used (Kilshaw and Annett, 1983).

### Materials and methods

#### Participants

A total of 76 healthy right-handed children were tested (19.4–5 year olds, 12 female, *M*_age_ = 4.73, *SD* = 0.5; 34.6–8 year olds, 12 female, *M*_age_ = 6.97, *SD* = 0.9; 23.9–12 year olds, 14 female, *M*_age_ = 10.83, *SD* = 1) and 36 healthy young right-handed adults (20 females *M*_age_ = 21.31, *SD* = 1). The handedness of the participants was determined utilizing the Waterloo Handedness Questionnaire. Prior to starting the study all participants were informed of the protocols and written consent was obtained. This study was approved by the Office of Research Ethics at Wilfrid Laurier University. One limitation of the study was that we were not able to obtain WHQ scores for some of the children due to their age as they may not fully understand the questions asked and may not provide reliable results.

#### Apparatus

The 22-item Waterloo Handedness Questionnaire (see Cavill and Bryden, 2003) was used to determine hand preference for the adults only. This study used two different types of pegboards: (1) The Lafayette Instrument (Model #32025) standard Grooved Pegboard, which will be referred to as the small pegboard from now on (see Ruff and Parker, 1993; Bryden and Roy, 2006); (2) Modified Grooved Pegboard, which will be referred to as the large pegboard from now on. The small pegboard has a 10.1 cm by 10.1 cm metal surface with 5 rows and 5 columns of grooved holes. The holes were aligned in a manner in which the peg must be carefully oriented in order to place the peg into the hole. All the holes were aligned in a different manner. Each participant was required to place 25 pegs (3.0 mm in diameter and 2.5 cm in length) into the receptacles. The large Grooved Pegboard is based on the design of the small pegboard and built to be approximately 2 times the size of the small pegboard. This pegboard has a 20.5 cm by 20.5 cm metal surface with five rows of 5 keyhole shaped holes with a receptacle at the bottom. The holes in this pegboard are positioned in the same way as the small pegboard. The pegs for this pegboard were scaled to a bigger size with a diameter of 9 and 70 mm in length.

#### Procedure

After acquiring informed written consent and determining handedness, the participants' thumbs and index fingers were measured. Participants were then asked to complete the two aforementioned pegboard tasks (small and large), in a randomized schedule. The Pegboard tasks require the participant to perform two different phases: place and replace; however for the purposes of this paper we will only focus on the place time. During the place phase, participants were asked to place pegs into their respective holes. For example, the participants were instructed to remove the pegs from the receptacle and place them in the grooves, starting on the side opposite to the hand placing the pegs. If the participant was starting with their right hand they would start on the left side and move to the right; whereas the left hand had the mirror image (started on the right side and move left).

Finally the participants were instructed to perform the task as quickly as possible. Timing commenced once the first peg was grasped and a total of three trials were performed by each hand for each of the pegboards.

#### Data reduction

Average movement time was calculated for each of the pegboards for all the groups. However, the data was not normally distributed; therefore a log base 10 transformation was applied for statistical analysis. The transformed data minimize the number of violations for statistical analysis (with the exception of the youngest children); therefore the transformed data was analyzed. The data was then transformed back for the purposes of presentation.

***Waterloo handedness questionnaire***. The questionnaire serves as self-report measure of hand preference, as participants were asked to indicate their preferred hand for 22-unimanual tasks (Steenhuis and Bryden, 1989). Each question permits five responses: “left always” (−2), “left usually” (−1), “uses both hands equally often” (0), “right usually” (+1), and “right always” (+2), thus enabling an overall handedness score to be computed by summing the responses. As expected participants averaged a positive score (adult females *M* = 31.6, *SD* = 5.6; adult males *M* = 28.9, *SD* = 3.9).

#### Data analysis

Place time for the children and adults was submitted to a 3 Group (younger children, older children, adults) × 2 Sex (male, female) × 2 Pegboards (small, large) × 2 Hand (left, right) mixed ANOVA with the last two factors as repeated measures. Any violations to the assumptions of normality were corrected using a Greenhouse-Geisser correction. *Post-hoc* analyses using Tukey HSD were used to examine any effects involving more than two means.

### Results

All main effects and interactions that were not of interest are presented in Table 1. A significant Sex × Pegboard interaction, *F*_(1, 104)_ = 8.4, *p* < 0.01, η^2^ = 0.08 was revealed. The *post-hoc* analysis, however, revealed that both females and males were better at the large pegboard (62.09 s, *SE* = 1.02 s and 59.43 s, *SE* = 1.02 s respectively) compared to the small pegboard (70.79 s, *SE* = 1.03 and 72.11 s, *SE* = 1.03 s respectively). However, *post-hoc* analysis did not reveal any differences between sexes on either pegboard.

**Table 1:** **Means and standard errors (in brackets) for main effects and interactions for both Experiments for all the variables (V)**.

**Exp**	**Effect**	**V1**	**V2**	**V3**	**V4**
**1**					
	Group	4–5	6–8	9–12	Adults
	*F*_(3, 104)_ = 82.97,	95.7 s	73.1 s	54.5 s (1 s)	49.6 s (1 s)
	*p* < 0.001 η^2^ = 0.71	(1.04 s)	(1.03 s)		
	Pegboard	Small	Large	N/A	N/A
	*F*_(1, 104)_ = 223.87,	71.5 s	60.8 s		
	*p* < 0.001, η^2^ = 0.68	(1.02 s)	(1.02 s)		
	Hand	P	NP	N/A	N/A
	*F*_(1, 104)_ = 172.24,	61.4 s	70.8 s		
	*p*< 0.001, η^2^ = 0.62	(1.01 s)	(1.02 s)		
	Group × Pegboard	4–5 year olds	6–8 year olds	9–12 year olds	Adults
	*F*_(3, 104)_ = 14.1,				
	*p* < 0.001, η^2^ = 0.29				
	Small Pegboard	103.99 s	77.8 s	57.02 s	56.36 s
		(1.04 s)	(1.03 s)	(1.03 s)	(1.03 s)
	Large	87.9 s	68.87 s	52 s	43.45 s
	Pegboard	(1.04 s)	(1.03 s)	(1.03 s)	(1.03 s)
	Group × Hand	4–5 year olds	6–8 year olds	9–12 year olds	Adults
	*F*_(3, 104)_ = 5.71,				
	*p* < 0.001, η^2^ = 0.62				
	Preferred	87.3 s	67.14 s	50.58 s	47.64 s
		(1.04 s)	(1.03 s)	(1.04 s)	(1.03 s)
	Non-Preferred	104.71 s	79.8 s	58.62 s	51.4 s
		(1.5 s)	(1.03 s)	(1.04 s)	(1.03 s)
**2**					
Pegboards	Group	YA	OA	N/A	N/A
	*F*_(1, 65)_ = 62.82,	53.7 s	76 s(1.89s)		
	*p* < 0.001, η^2^ = 0.49	(1.96 s)			
	Pegboard	Small	Large	N/A	N/A
	*F*_(1, 65)_ = 246.24,	73.3 s	56.4 s (0.95)		
	*p* < 0.001, η^2^ = 0.79	(1.77 s)			
	Hand	P	NP	N/A	N/A
	*F*_(1, 65)_ = 62.43,	61.5 s	68.2 s		
	*p* < 0.001, η^2^ = 0.49	(1.15 s)	(1.58 s)		
	Hand × Pegboard	Small	Large		
	*F*_(1, 65)_ = 14.44				
	*p* < 0.001, η^2^ = 0.18				
	Preferred	68.94 s	54.05 s		
		(1.49 s)	(0.93 s)		
	Non-Preferred	77.58 s	58.82 s		
		(2.18 s)	(1.09 s)		
Annett	Group	YA	OA	N/A	N/A
	*F*_(1, 65)_ = 61.9,	10.5 s	13.6 s		
	*p* < 0.001, η^2^ = 0.49	(1.02 s)	(1.02 s)		
	Hand	P	NP	N/A	N/A
	*F*_(1, 65)_ = 29.04,	11.6 s (1.02 s)	12.4 s		
	*p* < 0.001, η^2^ = 0.31		(1.95 s)		
Tapping	Group	YA	OA	N/A	N/A
	*F*_(1, 65)_ =6.93,	48.48 taps	42.84 taps		
	*p* = 0.011, η^2^ = 0.1	(1.5 taps)	(1.44 taps)		
	Sex	F	M	N/A	N/A
	*F*_(1, 65)_ = 5.42,	43.21 taps	48.12 taps		
	*p* = 0.023, η^2^ = 0.08	(1.5 taps)	(1.4 taps)		
	Hand	P	NP	N/A	N/A
	*F*_(1, 65)_ = 13.9,	46.82 taps	44.5 taps		
	*p* < 0.001, η^2^ = 0.18	(1.11 taps)	(0.98 taps)		

**Figure 1:**
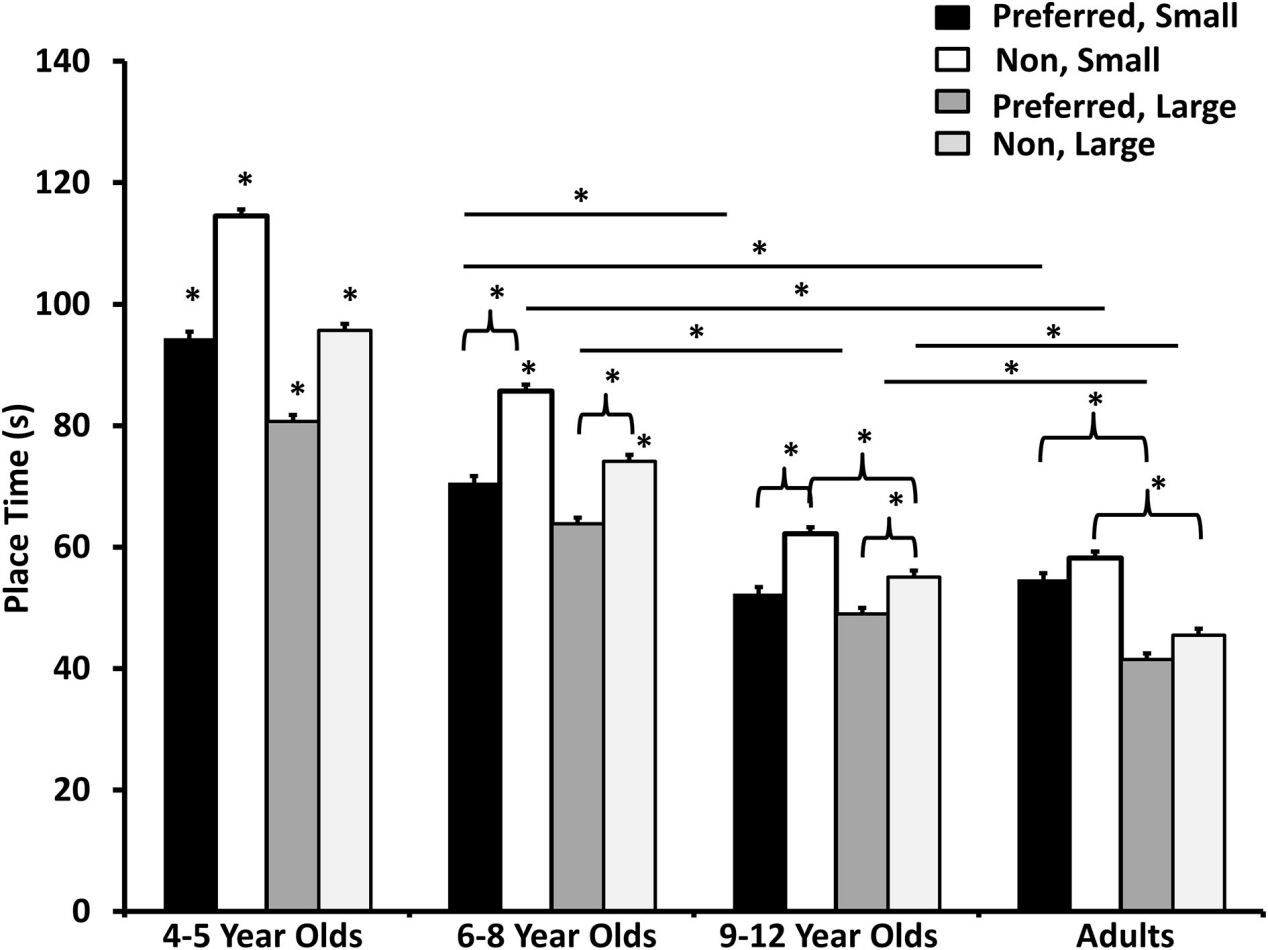
**Time taken to place all the pegs for all the groups using preferred and non-preferred hands**. Stars denote significant different from all others (no lines) or when directly compared to other conditions within a group (brackets) or between groups (lines).

A Group × Hand × Pegboard, *F*_(3, 104)_ = 3.03, *p* = 0.033, η^2^ = 0.08 was also revealed. Overall the youngest children (4–5) were slower compared to all other groups, regardless of hand used or pegboard size. The next slowest group was the 6–8 year olds, also showing hand differences regardless of pegboard size, and the 9–12 year olds and adults behaved similarly (see Figure 1). To further explain the trends, place time decreased as the age of the participants tested increased regardless of which pegboard size was used. Furthermore, asymmetries decreased as a function of age with the adults having little to no difference in how the preferred and non-preferred hands performed. Lastly, the youngest age group and the adults demonstrated faster place times when using both the small and large pegboards; however the 6–8 and the 9–12 year olds did not show a difference between the small and large pegboards when using their preferred hands (see Figure 1).

### Discussion

The results confirm one of our hypotheses, as the youngest children (4–5) were slower to complete the tasks (i.e., both pegboards) compared to older children (9–12). These findings are in line with previous findings of childhood performances (e.g., Gesell and Ames, 1947; Gooderham and Bryden, 2014). As well our findings demonstrate that the adults are least lateralized, as there were no statistical differences when comparing the preferred or non-preferred hands on both the Grooved Pegboard and larger pegboard tasks, which may be explained by previous findings on laterality. Researchers have proposed that as we age into our adult years, we have a reduced dependency on our preferred hand and rely more on biomechanical efficiency (Bishop et al., 1996; Bryden and Roy, 2006; Bryden et al., 2011; Gooderham and Bryden, 2014). However, it should be noted when the data for the adults are analyzed separately (not included in analysis with the data from the children; *F*_(1, 34)_ = 27.38, *p* < 0.001, η^2^ = 0.45 for main effect of Hand when adults are analyzed separately) the results demonstrate a difference between hands, therefore this suggests that the variability introduced by the other groups washes these differences out. Therefore, it can be said that the differences seen in the youngest participants, and the 6–8 year olds show a big performance difference between the hands, and these differences may attenuate as we age (as seen as gradual disappearance in hand difference until reach adulthood).

Our second hypothesis was generally supported as the youngest group performed the slowest regardless of which task was used. However, our finding suggest that children aged 9–12 behave more like adults and do not show performance difference between the hands contradictory to previous studies (Carlier et al., 2006; Rezaee et al., 2010; Gooderham and Bryden, 2014). It is important to note that we measured actual relative hand performance in movement time as opposed to preferred hand reaching used in these other studies, which may account for the difference in findings.

Lastly our hypothesis that sex differences would only be observed for the small pegboard was not supported. Instead no sex differences were found which we postulate may be related to the large differences in body sizes across children. That is, children are still growing therefore some hands for females may be bigger than males and vice-versa which may wash out any differences. In the future, it may be prudent to take more objective measures to account for hand size, such as the work by Peters et al. (1990), instead of having indirect measures, such as varying sizes of the pegboards, to account for hand sizes. Therefore, further exploration is warranted to determine if using the small and large pegboards would support previous findings when using older adults and if sex differences emerge after childhood. Therefore, the purpose of Experiment 2 was to further explore the aging process and determine whether or not manual asymmetries fluctuate.

## Experiment 2

For Experiment 2 we sought to further explore the aging process by examining how asymmetries may be affected past adulthood. As well, given that we failed to find any sex differences in Experiment 1, which we postulate might be related to differences in growth rates for males and females, we sought to explore the role of sex differences in manual asymmetries in adults and older adults in addition to further exploring the role of hand size. To explore hand size, we measured the size of the fingers in a similar fashion that used by Peters and colleagues (e.g., Peters et al., 1990; Peters and Campagnaro, 1996).

The specific hypothesis for Experiment 2 was that we would observe a Group by Hand interaction as the older group would revert back to child-like patterns of behavior, in that the difference between the performance of the preferred and non-preferred hands would decrease compared to the younger adults. The second hypothesis is that sex differences would disappear once finger size was used as a covariate and when the larger pegboard is used (see Peters et al., 1990) and when the large pegboard is used (Kilshaw and Annett, 1983). As well other measures of hand performance were used to determine if the findings of the small and large pegboard are task specific or can be generalized to other tasks such as the Annett Pegboard, peak grip strength, and finger tapping.

### Materials and methods

#### Participants

A total of 35 healthy young right-handed adults (15 female, *M*_age_ = 20.91, *SD* = 2.4) and 37 healthy older right-handed adults (20 female, *M*_age_ = 72.3, *SD* = 7.96) were tested. Prior to starting the study all participants were informed of the protocols and written consent was obtained. This study was approved by the Office of Research Ethics at the University of Waterloo.

#### Apparatus

The Waterloo Handedness Questionnaire (see Steenhuis and Bryden, 1989) was used to determine hand preference. This study used the same apparatus as in Experiment 1 with the addition of the Annett Pegboard, a dynamometer to measure grip strength, and a finger tapper to measure fine motor control as reflected in the number of taps.

#### Procedure

The same procedure as Experiment 1 was used, with the addition of the Annett pegboard in which participants moved the 10 pegs from the top row to the bottom row starting on the contralateral side. Upon completing the pegboard tasks participants utilized a hand dynamometer to measure peak grip strength (N) for each hand. Each participant utilized the dynamometer with each hand 3 times. Lastly, the participants had to perform a finger tapping task, where performance was measured by how many times a participant could tap a button in 10 s. Each participant performed the finger tapping (Lafayette) task 3 times with each hand. The order of tasks was randomized between participants.

#### Data reduction

Average movement time was calculated for the separate pegboard tasks, for the place component for each hand. As well the average grip strength was calculated for each hand, and the average number of taps for a 10 s period. For the Annett pegboard, the data was not normally distributed and therefore a log base 10 transformation was applied before statistical analysis and interpretation. The data was transformed back for the purposes of the presentation of results (i.e., following interpretation).

***Covariate analysis***. Finger size for the index finger and thumb were measured and summated in the same manner as Peters et al. (1990). However, instead of using separate covariates for the left and right hand measurements, principle component analysis was used to determine a representative covariate as the sizes of the fingers in both hands were highly correlated to each other (*r* = 0.91).

***Waterloo handedness questionnaire***. Here, participants were asked to indicate their preferred hand for 32-unimanual tasks (Steenhuis and Bryden, 1989). Each question permits five responses: “left always” (−2), “left usually” (−1), “uses both hands equally often” (0), “right usually” (+1), and “right always” (+2), thus enabling an overall handedness score to be computed by summing the responses. As expected participants average a positive score (younger females *M* = 46.87, *SD* = 8.3; younger males *M* = 40.3, *SD* = 13.85; older females *M* = 45.5, *SD* = 12.1; older males *M* = 46.8, *SD* = 9.9).

#### Data analysis

Place time for the small pegboard and large pegboard was submitted to a 2 Group (younger, older) × 2 Sex (female, male) × 2 Hand (left, right) × 2 Pegboard (small, large) mixed ANOVA, with the last two factors as repeated measures. Movement times for the Annett Pegboard, finger tapping, and hand grip strength were all analyzed separately in a 2 Group (younger, older) × 2 Sex (female, male) × 2 Hand (left, right) mixed ANOVA. Any violations to the assumptions of normality were corrected using a Greenhouse-Geisser correction. Any effects involving more than two means was *post-hoc* tested using Tukey HSD.

### Results

All main effects and interactions that were not of interest are presented in Table 1. Results are presented with hand size used as covariate with results prior to hand size being used as a covariate presented at the bottom of each section if there were any.

#### Small and large pegboard

There was a Group x Pegboard, *F*_(1, 65)_ = 19.22, *p* < 0.001, η^2^ = 0.23 interaction both younger and older adults are faster to complete the large pegboard (47.79 s, *SE* = 1.43 and 65.08 s, *SE* = 1.37 s respectively) compared to the small pegboard (59.75 s, *SE* = 2.64 and 86.95 s, *SE* = 2.54 s respectively). In addition, the younger adults were faster compared to the older adults on both the small and large pegboards.

A Sex × Pegboard, *F*_(1, 65)_ = 7.982, *p* = 0.006, η^2^ = 0.11 interaction was revealed. Females were faster at completing the small pegboard (69.41 s, *SE* = 2.62 s) compared to the males (77.11 s, *SE* = 2.52 s). However, there was no difference when comparing the large pegboard between sexes (55.78 s, *SE* = 1.41 s for females and 57.09 s, *SE* = 1.36 s for males).

**Figure 2:**
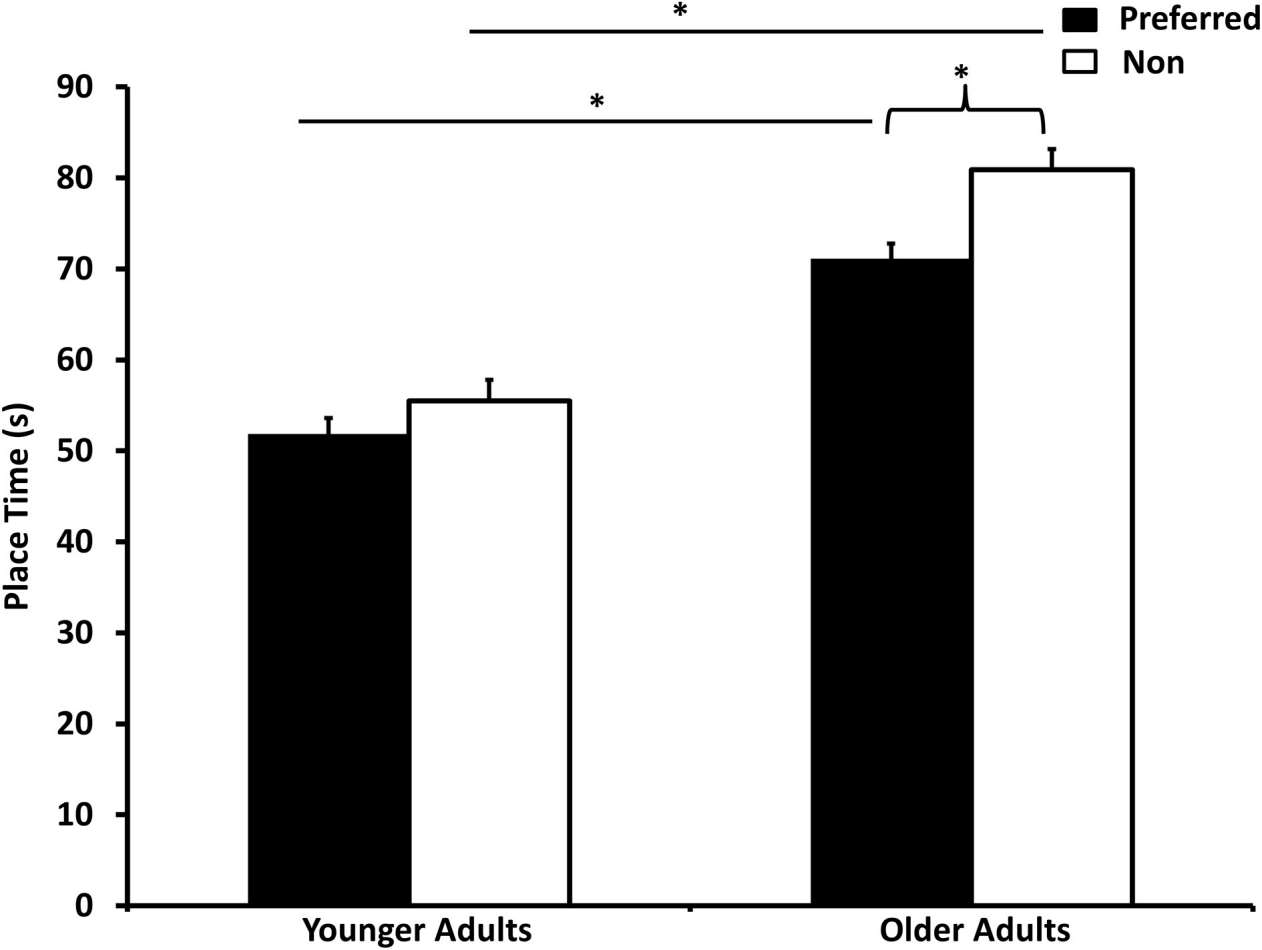
**Time taken to place the pegs using the preferred and non-preferred hands for both younger and older adults**. Between group comparisons are made using lines, and within group comparisons are made using brackets. Stars denote significant differences.

Finally the interaction of interest was the Group × Hand, *F*_(1, 65)_ = 11.63, *p* = 0.001, η^2^ = 0.15 interaction. There were no differences in the time to complete the tasks in the younger adults when comparing the preferred and non-preferred hands. However, the older adults took significantly longer to perform the tasks with the non-preferred hand (see Figure 2).

Analysis prior to using finger size as a covariate revealed a main effect for Sex (*p* = 0.023). The finding was that females (62.59 s, *SE* = 1.95 s) were slightly faster than males (67.1 s, *SE* = 1.87 s). However, after using finger size as a covariate, the main effect for Sex disappeared (*p* = 0.109).

#### Annett pegboard

A Hand × Group, *F*_(1, 65)_ = 5.38, *p* = 0.024, η^2^ = 0.08 was revealed. *Post-hoc* analysis showed that older adults did not show a difference between preferred (13.34 s, *SE* = 1.02 s) and non-preferred (13.8 s, *SE* = 1.02 s) hands. However, younger adults showed faster completion times with the preferred (10.05 s, *SE* = 1.02 s) compared to non-preferred (11.07 s, *SE* = 1.03 s) hand.

#### Grip strength

Only main effects were found for grip strength. A main effect for Group, *F*_(1, 65)_ = 52.61, *p* < 0.001, η^2^ = 0.45 was found, as the younger adults had a higher peak grip strength (36.2N, *SE* = 1.01N) compared to the older adults (25.7N, *SE* = 0.97N). There was also a main effect for Sex, *F*_(1, 65)_ = 52.16, *p* < 0.001, η^2^ = 0.45 was revealed. Males (36.1N, *SE* = 0.97N) had higher peak grip strength compared to females (25.8N, *SE* = 1N). Lastly a main effect for Hand, *F*_(1, 65)_ = 31.55, *p* < 0.001, η^2^ = 0.33, was revealed as the preferred hand (32.2N, *SE* = 0.71N) had higher peak grip strength compared to the non-preferred hand (29.7N, *SE* = 0.71N).

#### Finger tapping

A Hand × Group × Sex, *F*_(1, 65)_ = 4.55, *p* = 0.037, η^2^ = 0.07 was found. When *post-hoc* analysis was done, younger males (51 taps, *SE* = 1.9 taps) were able to tap more than younger females (43.47 taps, *SE* = 2.1 taps) when using their preferred hand. However, when using their non-preferred hands younger males and females did not differ (50.76 taps, *SE* = 2.1 taps and 48.07 taps, *SE* = 2.37 taps respectively). In addition younger males were able to tap more with their preferred hands compared to older males using their preferred hands (43.68 taps, *SE* = 2.11 taps) but did not differ when comparing their non-preferred hands (46.95 taps, *SE* = 2.4 taps). For females, the opposite was true as the younger adults were able to tap more with their non-preferred hands compared to the older females (41.53 taps, *SE* = 2.1 taps) but not when comparing the preferred hands (39.2 taps, *SE* = 1.9 taps for older females). The older adults did not differ when comparing preferred or non-preferred hands or sexes.

### Discussion

When aging is further explored when using the Grooved pegboards, it seems that manual asymmetries increase as a function of aging, which supports the hemi-aging model. Younger adults again did not show any differences but the older adults demonstrated better performance using the preferred hand. However, the findings of the Annett Pegboard support a decrease in manual asymmetries, or the HAROLD model or the use dependent plasticity model. Our findings cannot determine whether the motor performance of the non-preferred hand improved or the performance of the preferred hand declined for the older participants, instead future research may need to conducted with a longitudinal design rather than cross-sectional. Furthermore, finger tapping revealed that younger males were able to tap more than females when using their preferred hands and that older adults did not show any sex or hand differences. Therefore, it seems that our findings suggest a degree of task specificity in which different tasks produce different findings as to whether or not laterality continues as we age.

When finger size was used as a covariate most sex differences disappeared; however, a sex difference still remained when comparing the different pegboards. This suggests that even when using the small pegboard and accounting for finger size, females are still better than males. Our findings do not show the same results as those of Peters et al. (1990) that sex differences disappear after hand size is taking into consideration. The difference between our results and previous literature is discussed in the general discussion. In a related finding, our findings support those of Kilshaw and Annett (1983) as sex differences disappeared once the large pegboard was used.

## General discussion

Laterality and more specifically asymmetries are studied in different situations and throughout the lifespan (e.g., Gesell and Ames, 1947; Gooderham and Bryden, 2014). Since the pioneering days of Woodworth (1899) different theories have been proposed to understand how manual asymmetries may change with age (e.g., the hemi-aging model, hemispheric asymmetry reduction, and HAROLD model). Each of the models are informative within their own right, however our data best supports a mixture of these models. When the task is a pegboard placing task, children tended to have longer completion times and the times decreased as a function of age (Experiment 1). When the pegboard tasks were used for older adults it seems that they reverted back to child-like performance (Experiment 2) in that the differences in hand performance were demonstrated again. These findings seem to support the hemi-aging model.

The overall view of Grooved Pegboard task in our study seems to suggest that laterality is present at a very early age, then decreases into adulthood, and finally increases again as we age further. However, the limitation of aging research is that most is done using cross-sectional methods which does introduce sampling errors as we cannot definitively say that children who show laterality will grow and still demonstrate laterality once they are over the age of 60. Perhaps biomechanical efficiency (e.g., Bishop et al., 1996; Bryden and Roy, 2006; Bryden et al., 2011; Gooderham and Bryden, 2014) does play a role in determine which hand will be used, and therefore may influence hand performance. Although it may be the case that once we age further, 60+, we may be more confident using our preferred hand and once again forgo biomechanical efficiency and instead use our preferred hand.

One of the strengths of these studies is that the same participants were tested in multiple hand performance tasks to determine if global asymmetries exist for dexterity tasks. This design has allowed us to see that laterality is task specific, in that the Annett pegboard did not show the same finding as the Grooved Pegboard. Rather the HAROLD or the use dependent plasticity models were supported for the Annett Pegboard. Finally the findings of the grip strength demonstrate support for those of Gooderham and Bryden (2014) in stating that hand asymmetries do not change as we become older. Therefore, the question remains, what model is best for understanding the relationship between aging and manual dominance? It seems that asymmetries are task dependent in that different tasks reveal different trends.

By tearing apart the differences in tasks we may begin to understand the different trends associated with aging. The Grooved Pegboard and large pegboard require the participants to grasp a peg that is near them and place the peg into a receptacle that is further away from the participant. Furthermore, the participants are often required to rotate the peg in their fingers in order to successfully place the pegs in the receptacles for the Grooved Pegboard. However, when using the Annett Pegboard the participants grasp the peg starting away from the body and place the peg in a receptacle that is closer to their body. Think about these task differences with reference to the aiming literature. Here it has been revealed that moving the hand away from the body differs from moving it toward the body (Lyons et al., 2006; Heath and Binsted, 2007). The pointing movements toward the body were faster and less variable which supports the findings of Lyons et al. (2006) that aiming toward targets that are closer produces better accuracy. Going back to our results, perhaps differences in asymmetries may exist by changing the location of the receptacles (further or closer to the participant) which may differ in how accurate our movements may be. Therefore, placing a peg that already requires more accuracy (Grooved Pegboard compared to Annett) in a receptacle further from the body may challenge the perceptual-motor system more than placing a peg in a receptacle closer to the body. Our recommendation is that when using different tasks in the future, the different movements that are required to complete the tasks should be considered (see Gooderham and Bryden, 2014) and how directionality or task precision may play a factor.

One other hypothesis that we had was that the size of the fingers would account for sex differences in performance. In previous research (e.g., Peters et al., 1990; Peters and Campagnaro, 1996) finger size was revealed to be an important covariate, however, our data did not fully support this finding. Indeed some of the sex differences did disappear after using finger size as a covariate (Experiment 2), however a few interactions involving sex remained. Specifically females were still better than males when performing the Grooved Pegboard task. Therefore, the differences in performance were not fully accounted by the difference in finger size; rather there are other factors that may account for the sex differences. We must point out, however, that the experimental procedure of Peters et al. (1990) was slightly different than ours. For example, we had individuals perform the tasks with both left and right hands and to incorporate the finger size as a covariate we used a principle component analysis so as to have one covariate measure. This differed from Peters et al. (1990) as only one measure, the right hand, was used for their analyses. Furthermore, when the large pegboard was used, sex differences disappeared which support the findings of Kilshaw and Annett (1983). Therefore, perhaps once individuals' bodies have finished growing the size of the pegs may affect performance differently for males and female. As well perhaps by using the non-dominant arm, the left hand in our experiment, sex differences may be larger and show more of an advantage for females for fine dexterity tasks.

## Conclusion

It seems that manual asymmetries are a product of aging, as young children explore the world using both hands. It is not until later on that we prefer to use one hand more than the other, and to a point that it may be exclusively used even for contralateral reaching during adolescent years (e.g., Carlier et al., 2006). However, the question whether or not we revert to child-like behavior as we age is not clearly answered. Our results suggest that there were task specific manual asymmetries, as manual dexterity and accuracy requirements may tax the motor systems more in tasks like the Grooved Pegboard and thus reveal preferred hand dominance, while other tasks like the Annett Pegboard do not. Furthermore, the Grooved Pegboard and the use of the non-dominant hand may enhance the differences in sexes, revealing sex differences above that accounted for with finger size; therefore, we a need to standardize methods to get a better sense of lateralization throughout the lifespan. Instead of discussing if asymmetries occur or do not occur, perhaps both occur and we should focus on examining which underlying processes are preserved and which deteriorate with age.

### Conflict of interest statement

The authors declare that the research was conducted in the absence of any commercial or financial relationships that could be construed as a potential conflict of interest.
